# How does an organism extract relevant information from transcription factor concentrations?

**DOI:** 10.1042/BST20220333

**Published:** 2022-09-16

**Authors:** Marianne Bauer

**Affiliations:** 1 Bionanoscience Department, Delft University of Technology, van der Maasweg 9, 2629 Delft, The Netherlands; 2 Joseph Henry Laboratories of Physics, Princeton University, Princeton, NJ 08544, U.S.A.; 3 Lewis–Sigler Institute for Integrative Genomics Princeton University, Princeton, NJ 08544, U.S.A.

**Keywords:** biological models, biophysics, gene expression and regulation

## Abstract

How does an organism regulate its genes? The involved regulation typically occurs in terms of a signal processing chain: an externally applied stimulus or a maternally supplied transcription factor leads to the expression of some downstream genes, which, in turn, are transcription factors for further genes. Especially during development, these transcription factors are frequently expressed in amounts where noise is still important; yet, the signals that they provide must not be lost in the noise. Thus, the organism needs to extract exactly relevant information in the signal. New experimental approaches involving single-molecule measurements at high temporal precision as well as increased precision in manipulations directly on the genome are allowing us to tackle this question anew. These new experimental advances mean that also from the theoretical side, theoretical advances should be possible. In this review, I will describe, specifically on the example of fly embryo gene regulation, how theoretical approaches, especially from inference and information theory, can help in understanding gene regulation. To do so, I will first review some more traditional theoretical models for gene regulation, followed by a brief discussion of information-theoretical approaches and when they can be applied. I will then introduce early fly development as an exemplary system where such information-theoretical approaches have traditionally been applied and can be applied; I will specifically focus on how one such method, namely the information bottleneck approach, has recently been used to infer structural features of enhancer architecture.

## Theoretical approaches for gene regulation

**Figure 1.**
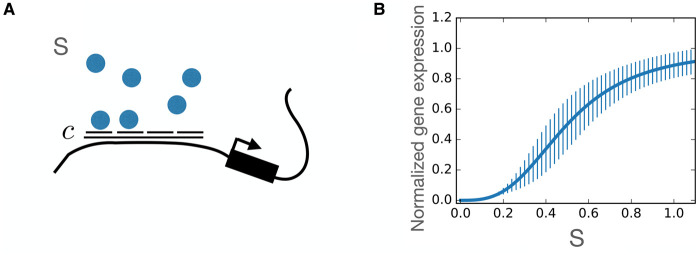
Gene regulation as a continuous function of number of bound transcription factors. Left: Sketch for binding sites for transcription factors (blue) of concentration *S* close to a gene's promoter; right: expression as depending on S can be modeled by a Hill-function.

The discovery that *Escherichia coli* could switch from growing on glucose to lactose depending on lactose’s presence in the environment showed that cells can respond to environmental stimuli by gene regulation [1]. As the lac-operon was one of the most impressive early discoveries in gene regulation, many early models for gene regulation, also in the context of development, were based on it [2]: the lac gene is regulated by transcription factors whose binding sites are in direct proximity to the gene’s promoter (see Figure 1). The area around the promoter contains binding sites for transcription factor molecules that can facilitate or block binding of the polymerase and, therefore, activate or repress the expression of the gene [3, 4]. A model for this assumes that h transcription factors of concentration s bind to the binding sites (cooperatively, i.e. at the same time), and that the mean concentration of expressed output (assuming ergodicity) corresponds to the averaged probability of the bound state. The chemical master equation for this process reads,

1 $$\frac{dP(0,t)}{dt}=k_{\text{off}}-(k_{\text{off}}+k_{\text{on}}s^{h})P(0,t),$$

where $k_{\text{on}}$ and $k_{\text{off}}$ are the rate constants for binding and unbinding and P(0,t) the probability that the site is free at time t. Then, in steady state, the mean presence of the bound state is

2 $$\bar{c}=\lim_{T\to \infty }\frac{1}{T}\int_{0}^{T}(1-P(0,t))\;dt=\frac{s^{h}}{k_{\text{off}}/k_{\text{on}}+s^{h}}.\;(2)$$

This is a Hill-function (see Figure 1) [5, 6]: gene expression increases sigmoidally with transcription factor concentration and the steepness of the increase is given by h.

**Figure 2.**
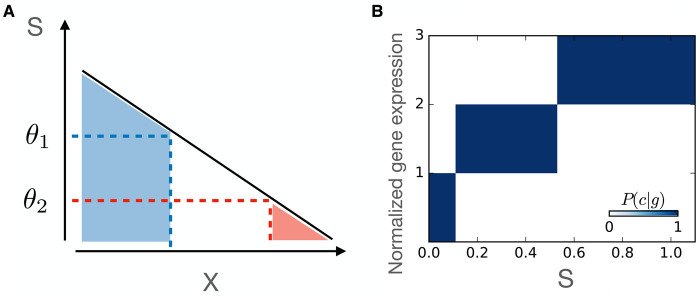
Gene regulation as a threshold-like response to varying transcription factor concentrations S. Left: French flag model where regulatory response is sharply different if S exceeds a particular threshold (here for thresholds $\theta_{1}$ and $\theta_{2}$; right: gene expression of a single gene as a function of *S* for these two thresholds.

Another canonical model for gene regulation is based on sharp thresholds: this idea originated from Wolpert [7] concerning the question of how cells can express different genes given different transcription factor concentrations. Here, a gene is expressed (at maximal level) when the concentration of a particular input transcription factor is higher than a certain value, and not expressed (g=0) when the transcription factor concentration is lower than this value; for a transcription factor gradient in development, for multiple genes, this corresponds to the so-called ‘French flag model’, see Figure 2 [8, 9]. This response to gene expression based on a sharp threshold value of concentration can be phrased mathematically as

3 $$C=H(s-\theta_{1}),\;(3)$$

where H is the Heaviside stepfunction, defines as H(x)=0 if x<0 and H(x)=1 if x>0; for multiple thresholds, this works analogously, i.e. the amount of expressed genes varies in discrete units or states shown in Figure 2 (right). Mechanistically, one could assume that such thresholds can be implemented in terms of a very steep Hill’s function, with h→∞.

The advantage of these models is their simplicity, or their usefulness as a ‘limiting case’: for example, the analysis of the graph-theoretical models has shown that the Hill function from equation (2) gives the steepest possible slope (or threshold) of all various individual combinations of transcription factor binding [10, 11]. Thus, both of these models are still frequently used for understanding gene expression [12–14].

Yet, one important change in thinking about gene regulation around the early 2000s was the focus on noise and stochasticity of gene expression [15]. This stochasticity is a consequence of both stochastic promoter bursting and the limited number of transcription molecules which bind to the binding site region in a limited amount of time.

On the theoretical side, calculations for noise originate from work in 1977 on chemotaxis (which involves the sensing of a molecular gradient by receptors) [16]: Berg and Purcell argued that the signal-to-noise ratio δc/c with which a 1D receptor of size A which measures for time τ can infer the concentration s of a freely diffusing signal molecule (diffusion constant D):

4 $${\left(\frac{\delta c}{c}\right)}^{2}=\frac{2}{Das\tau (1-p)},\;(4)$$

where p describes the occupation at the binding site region (the term thus implies that binding can not occur when the binding site is already fully occupied). Here, we use c in order to make explicit that this denotes the cell’s estimate of the signal s.

Remarkably, this limit presents still a lower bound for noise in binding in equilibrium. More general work in the early 2000s [17], which included account the noise of binding and unbinding of the molecules, showed that the signal-to-noise ratio of inferring c changes to [17, 18]

5 $${\left(\frac{\delta c}{c}\right)}^{2}=\frac{2}{Das\tau (1-p)}+\frac{2}{k_{\mathrm{on}}s(1-p)\tau };\;(5)$$

the first term corresponds to the diffusion-limited contribution (c.f. 4), and the second term to noise from binding events. This signal-to-noise ratio is higher than equation (4). Similarly, extensions towards cooperative binding [19] did not yield a lower bound. Clever readout [20] or spending energy (likely during gene regulation) [21] can lower this bound. Nevertheless, from a modeling perspective, the equilibrium case is frequently preferred as it depends less on the details of the model.

While the Berg–Purcell bound can be applied to the Hill-function model with a single binding site, generalization to more binding sites or more complicated mechanisms is difficult. The thresholded model does not incorporate noise at all: this is a key shortcoming of this intuitive model, especially as it has been suggested that increased cooperativity (i.e. a more threshold-like mechanism) may raise the noise by increasing the correlation time of the input noise, impeding noise averaging [19, 22].

The above discussion already shows that it is difficult to calculate both the mean and noise of gene expression in a model bottom-up. In addition, there are a series of experimental insights since the early work on gene regulation, which make the situation even more complex.

## Gene regulation now

**Figure 3.**
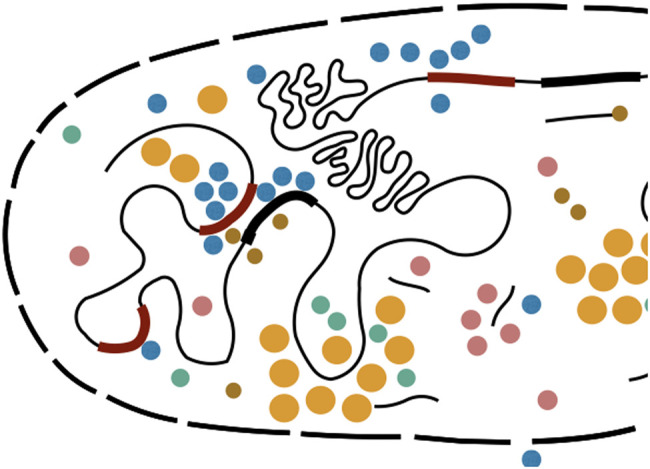
Sketch of gene regulatory environment: several enhancers (dark red) can regulate a gene (dark black); protein concentrations can be inhomogeneous.

In eukaryotic organisms, the regulatory architecture is different from the lac-operon: genes can be regulated by one or more promoters, as well as several regions with binding sites for transcription factors (the so-called enhancers) which can be several kilobasepairs away from the promoter or the gene [23] (sketch in Figure 3). These enhancers frequently have binding sites for a larger number of different transcription factors, some of which have pioneering activities that make the chromatin accessible.

Modifications of these models to incorporate the more complex regulatory landscape of individual transcription factor binding have, for example, been made by the so-called ‘thermodynamic models’ for transcription [24, 25]. Here, the probability of the downstream gene to turn on and off depends on a partition function, which takes into account the probability of various combinations of bound states of transcription factors to binding site regions close to the DNA given the binding energies; different such combinations can lead to different levels of gene expression. Recently, how the binding of transcription factors is affected when other transcription factors are already bound has been investigated by graph-theoretical models for transcription factor binding [11]. Finally, ‘kinetic’ models for gene regulation have taken seriously the possibility that not the thermodynamic steady state, but a series of non-equilibrium reactions are responsible for gene regulation [26–28]; these kinetic models are particularly important with the recent trend to investigate the importance of pioneer transcription factors which make chromatin accessible in the first place [29–34]. While these models present a significant progress, incorporating the effects of the joint activity of several enhancer elements is difficult. In addition, the calculation of noise outside a strict thermodynamic framework is difficult and highly parameter specific.

The situation is further complicated by the idea that transcription may involve a topological change in the genome that changes enhancer-promoter distances [34–37]. One additional complication is derived from the recent research focus on cellular compartmentalization, which means that the concentrations of transcription factors may vary across the cell [38, 39]. This is especially topical now as liquid–liquid phase separation (LLPS) has recently been implied to also affect transcription [40–43]. While LLPS is being established as a mechanism for cellular compartmentalization when the numbers of involved proteins are large, to what extent it affects gene regulation is still intensely debated [44]: especially in development, concentrations of some transcription factor peak of order 10 000 molecules per cell [30, 45–47]; thus, even if only ca 50 inhomogeneities or droplets are observed, they would need to contain less than the LLPS-typical numbers of 100s of molecules per droplet if only these transcription factors make up the droplet; this makes the applicability of the mechanism difficult. Nevertheless, the fact that transcription factors are likely inhomogeneously distributed is gaining prominence in the field [29, 48, 49].

These heterogeneous transcription factor distributions matter from the modeling perspective: frequently, transcription factor concentrations are only available averaged across the entire cell, but the local concentration of the transcription factor close to its binding site is required for the model (see equation 2). If these concentrations are unknown, estimating parameters for more specific models might lead to flawed conclusions. Similarly, calculating the noise of binding at the binding site regions is almost impossible when neither the number of transcription factors nor the mechanism for their accumulation around the binding site is known.

Overall, the added experimental complexities mean that although many advances have been made regarding modeling the regulation of individual genes in specific developmental time periods, an overall conceptual picture is still lacking. Such a conceptual picture is nevertheless important: conceptual understanding can help predict whether a particular gene may have many enhancers, where they might be located, or what binding site arrangements can detrimentally change expression.

Thus, in the following section, I will introduce a ‘top-down’ approach to complement to the ‘bottom-up’ mechanistic models; this approach is based on data and attempts to infer structural features necessary for precise gene regulation from these data.

## Sensing approach to gene regulation

A complex system where it has been similarly complicated to draw up simple models due to the large number of functional elements involved is a net of neurons, such as the brain. One can think of the Hill functions, or more specific molecular schemes, as being like the Hodgkin–Huxley model for the electrical dynamics of neurons [50]. An alternative is to take the result that these dynamics generate action potentials or ‘spikes’, and ask how these spikes represent information of relevance to the organism [51]. The hope is that there are principles governing this representation without reference to molecular details. This question of ‘reading the code’ thus makes use of ideas from statistical physics or network theory and also from signal processing [52–55]. This approach has had considerable success in the neural context, and we can hope that something similar will help us think about information flow through transcriptional regulation.

One particularly exciting approach here is to treat the interpretation of the transcription factor concentration(s) as a (combinatorial) sensing problem. Such ‘efficient’ sensing approaches have been successful in neuroscience, for example, concerning olfaction [56] or concerning photoreceptors [57]. A crucial starting point for information optimization in neuronal systems was the work by Laughlin [51, 58]: he argued that photoreceptors are assigned such that they pick up on the most informative part of the signal, so that they can extract the most possible information given a limited number of receptors. This structural knowledge is important also for extracting information from transcription factor concentrations: given the typical statistics of the transcription factor signal, the hope would be to infer how many sensors are necessary to provide a certain amount of information and how they should optimally be distributed to extract this.

Before briefly introducing the information-theoretic optimization in the sensing problem introduced by Laughlin as an example of a signal processing optimization, I want to emphasize one difference between electronic signal transfer and biological systems: In electronic signal transfer, one can consider how to best represent (source coding: optimizing entropy), and how to best transmit a message (channel coding: optimizing error correction). In biological systems, it can be difficult to differentiate the signal from what the message should be (for example, for signal processing of photoreceptors in the eye, the intuition could be that the set of messages is the set of maximally distinct images; however, some animals may care less about specific features of the images). In addition, in the processing of gene regulatory signals, the source (chemical concentration) and the channel characteristics (noise profiles) can be modified biologically (i.e. have evolved evolutionarily). Thus, it is not a priori helpful to think of coding categories as having been separately optimized in a joint source-channel coding sense [59], but better to use one’s biological intuition to investigate a plausible optimization goal, and see what one learns. In the spirit of not distinguishing signal-processing categories, I will, in the following, introduce Laughlin’s sensing problem phrased in terms of an optimization of information (c.f. [51]), rather than entropy.

Laughlin was wondering how a class of insect eye neurons, the so-called large monopolar cells (LMCs) can sense light intensity from different natural landscapes. We can denote the light intensity or signal by J, which can increase from 0 to a particular value $J_{\text{max}}$; the distribution of different intensities is P(J). The information provided in this signal needs to be transferred by the LMCs in terms of a graded potential, meaning that the LMC integrates the signal intensity from a series of photoreceptors [60] and uses the value of this potential as a proxy for the value of the intensity. We call this interpretation of the signal C (we will discuss this in more detail later). We now want to optimize the mutual information

6 $$I(J;C)=\iint dJ\;dCP(J,C)\log_{2}\frac{P(J,C)}{P(J)P(C)},\;(6)$$

where P(J,C) is the joint probability distribution and P(C) the marginal distribution of the graded potential. Optimizing the mutual information corresponds to essentially maximizing the correlations between J and C. We note that the mutual expression can also be expressed as

7 I(J;C)=H(C)−H(C|J),(7)

where

8 H(C)=−∫dCP(C)log⁡P(C)(8)

and H(C|J) are the entropy and conditional entropy, respectively. The mutual information is at its maximal value, the entropy of C, when J and C are maximally correlated. We want to find an encoding J→C that maximizes this mutual information; this encoding can be written as a function that assigns one or many values of potential C (depending on the noise in the encoding) to a value of light intensity J. It is important to note that we will not be able to calculate what numerical value C should have for any particular J: from a decoding perspective, it is irrelevant if high light intensities should be encoded by a low potential or by a high potential, as long as the mapping is clear.

To make the problem simple, we assume that the noise in the encoding J→C is very low. Often, we can assume that the encoding J→C has a probabilistic encoding in which C has a Gaussian distribution around a mean, $\bar{C}=f\;(J)$; this means that

9 $$P(C\;|\;J)=\frac{1}{\sqrt{2\pi \sigma^{2}}}\exp\left[-\frac{(C-\bar{C}{)}^{2}}{2\sigma^{2}}\right].\;(9)$$

Then, we can calculate the probability distribution P(C) analytically and relate it to the inverse of the derivative of $\bar{C}$ with respect to J, $P(C)=|df/dJ{|}_{J_{0}}^{-1}P(J)$ [51, 61]. Thus, optimizing the mutual information over all possible encodings $\bar{C}=f\;(J)$ for Gaussian distribution corresponds to, modulo normalization factors, optimizing over all possible probability distributions P(C).

If the noise σ is lower than a reasonable discretization of C (i.e. if we think the graded voltages can only be resolved to a certain value), we can assign a single value C to each value J. Then, the conditional entropy is zero. Thus, in Laughlin’s case, maximizing the mutual information corresponds essentially to maximizing the entropy in the encoding variable C. Maximizing the entropy is a simple information-theoretic problem, which can be solved using the method of Lagrangian multipliers [59]: the distribution P(C) that maximizes the entropy is the uniform distribution. Since we now know that $P(C)=\text{const}=|df/dJ{|}_{J_{0}}^{-1}P(J)$, we can see that df/dJ=P(J). This means that the best possible encoding for light intensities is one where the slope of the encoding matches the distribution over typical light intensities that typical insects see. This information-theoretic result is exactly what Laughlin found in his data [58].

In the next section, we will apply this sensing optimization to transcription factors.

## Sensing applied to transcription factors

We note that similarly to the light intensity, we assume that the ‘intensity’ or concentration of the transcription factor provides relevant information to cells. Especially in early fly development, the concentrations of certain transcription factors, such as the maternal morphogen Bicoid, provide information about a particular cell’s fate [62]: cells close to the head of the embryo at high bicoid concentration differentiate differently from cells close to the tail end of the embryo. In fact, neighboring cells along the embryonal axis distinguish almost uniquely into different cell fates (e.g. different body segments, such as thorax and abdomen, regulated by the hox genes, and even within a segment, different pair-rule genes are expressed at different concentrations). Cells will need to read the transcription factor concentration signal in a way that maximizes the information between the signal and the future cell fate. For simplicity, we can label the cell fate by its position along the embryonal axis, X. This idea goes back to Wolpert’s idea about ‘positional information’, as in the French flag model introduced above, but was developed and made more precise in a series of papers by Bialek, Gregor, Wieschaus and colleagues [45, 61, 63, 64].

**Figure 4.**
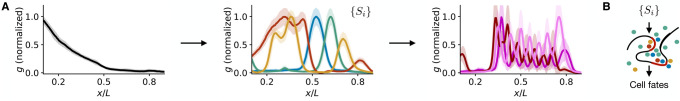
**Sketch for signal processing in the fly embryo.** (**A**) Expression patterns of the maternal Bicoid gradient, which regulates the four gap genes, which, in turn, regulate the seven pair-rule genes (three shown). The pair-rule genes, together with the hox genes, determine the fly’s segmentation along the embryonal axis, X, or its cell fates. Data from [45, 64]. (**B**) Gap gene expression patterns present signals that need to be interpreted by the gene regulatory apparatus for the correct cell fates differentiation.

In the case for fly development, there is thus a clear variable we care about (cell fates along the embryonal axis X). The signals that provide information about this cell fate are the four so-called gap genes (see Figure 4): they are expressed just downstream of Bicoid and two other maternally supplied signals. They form a complete set of inputs, because their expression profiles provide enough information about the downstream cell fates [63], and this information can be used to predict the expression pattern of the downstream pair-rule genes [64]. Thus, for the question of how to extract information from transcription factor signals, we have, in the fly embryo, a clear set of candidate signals where we can investigate whether and how information can be extracted, based on data.

The complication compared with Laughlin’s example in the previous section is that the signals and the variable, we care about, are different. In Laughlin’s example, the intuition was that the different intensities J were the signals that needed to be maximally distinguished. Here, the gap gene expression concentrations are signals $\left\{S_{i}\right\}$ for i∈{Hb,Gt,Kr,Kni} to represent the four genes *Hunchback*, *Giant*, *Krupel* and *Knirps*, and the cell fates of the set of possible positions along the axis, X. We are *not* interested in optimizing I(S;X) (which would correspond to designing a set of signals, {S}, which maximize the different responses or cell fate decisions along X). Instead, we take for granted the shape of the signals or gap expression profiles, and we are interested in how this biological signal can be interpreted by the cell in order to learn about the future cell’s fate. Thus, what we need to optimize is the cell’s reading of the signal (see Figure 4B); we denote this measurement or interpretation by C, which stands for compression, as a compression can be seen as an efficient measurement of the signal. In other words, we want to maximize the information that cells have, after their measurement of the signal, about their future cell fate decisions, i.e. I(C;X). We know that this reading or measurement is noisy, because of the stochastic noise with transcription factor arrival and binding discussed above. If we knew the mechanism for binding and arrival, we could calculate the probability distribution P(C|S), i.e. the cell’s internal measurement for every value of the signal, and then we could calculate I(C;S). However, we do not want to make an assumption about this mechanism; instead, we want to capture the essence but not the details of the limitations in any mechanism. Thus, we maximize I(C;X) for various values of I(C;S), or for various values of noisy measurements. For each value of I(C;S), we want to infer the encoding P(C|S) that extracts the most information about X; we can then compare this to calculations of I(C;S) and I(C;X) from various mechanisms. This inference will allow us to see how the cell would optimally set up the measurement if it had to be noisy.

This optimization procedure can be phrased as the optimization goal

10 $$\max_{P(C\;|\;S)}I(C;X)-\lambda I(C;S).\;(10)$$

This optimization goal corresponds to the information bottleneck optimization goal [65]; for this optimization goal, an analytic expression for P(C|S) exists that allows one to calculate P(C|S) self-consistently for each value of λ, and, due to the numerical discretization, for each value of discrete levels of C. According to this self-consistent equation, P(C|S) reads.

This information bottleneck algorithm is a compression algorithm that has recently enjoyed an increase in popularity, due to interest from machine learning [66, 67]: in image recognition, one is also interested in compressing away aspects of an image that do not contribute to our recognition of it. Similarly, the question when extracting transcription factor signals is which aspects of the signal are most informative, so that the organism can concentrate on sensing them more precisely. To go towards continuous C, we can simply ensure that the number of discrete levels of C is large.

**Figure 5.**
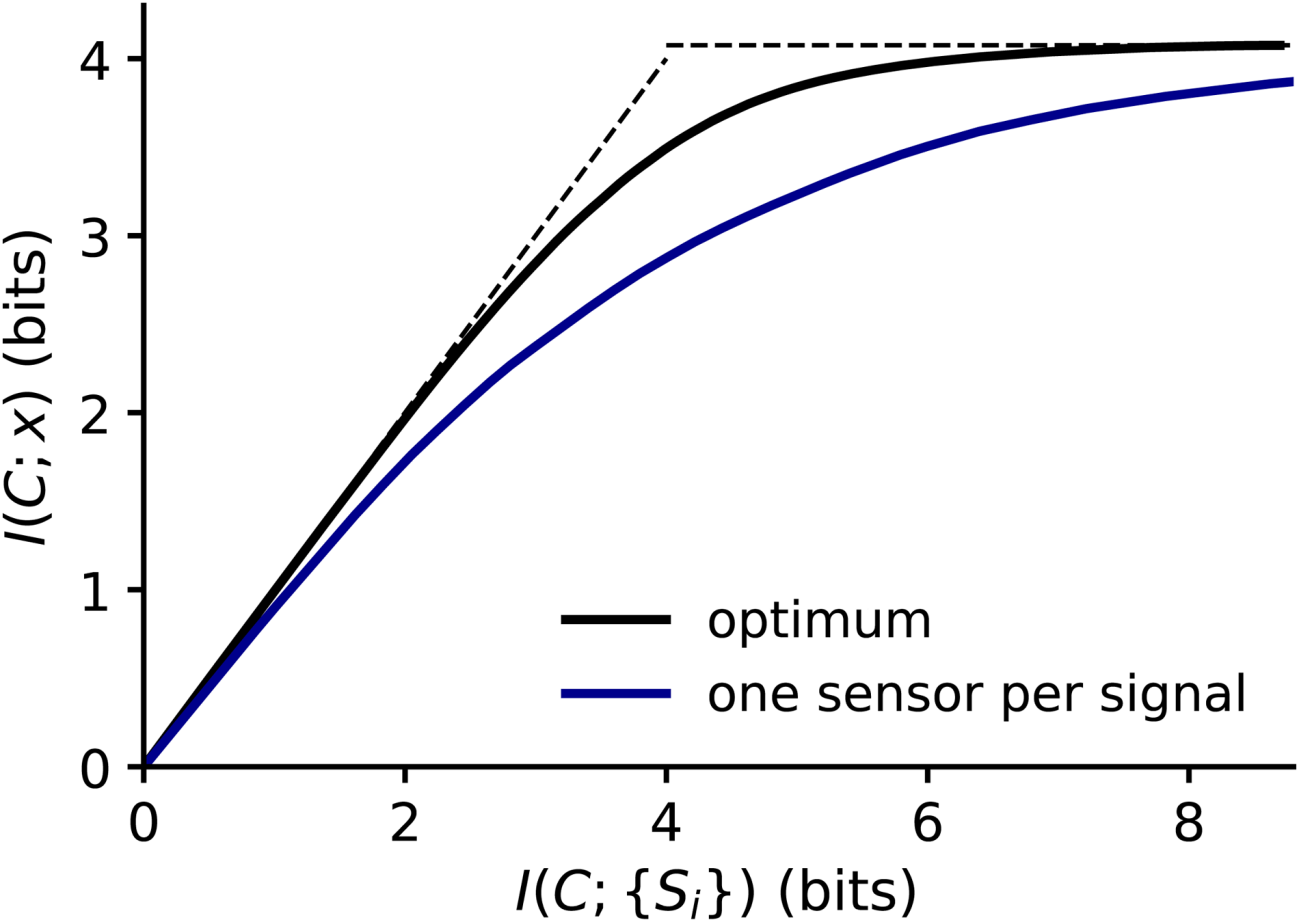
**The optimal bottleneck curve for a single sensor for all genes (black) and with four sensors optimized for all genes separately (blue).** The x-axis shows the information capacity of the sensor and the y-axis the information that the sensor has cell fates, which we want to maximize. Data replotted from [68].

We performed this calculation in [68], and I briefly summarize the key results here. For example, we can look at how much I(C;X) we can obtain at best for each value of I(C;S). We show this optimal trace in the information plane (where we plot I(C;S) on the x and I(C;X) on the y-axis) in Figure 5. We note that all possible values on the information plane are below the diagonal (where I(C;X)≦I(C;S)) and below the top dashed line (where I(C;X)≦I(S;X)). This top line is at I(S;X)≈4.1bit, which is the amount of information that the gap genes provide about cell fates [63]. This upper bound is due to the data processing inequality: effectively, it means that the cell can never obtain more information by its measurement than the signal provides. The optimal sensing bound calculated by the bottleneck algorithm is close to the best possible bounds, in that it increases quite steeply along the diagonal initially. This is not necessarily the case when one tries to find the best possible signal processing from a set of neurons [69], and suggests that the gap transcription factors here really provide a complete signal that can be sensed well.

## What do enhancers need to do if they extract signals optimally?

**Figure 6.**
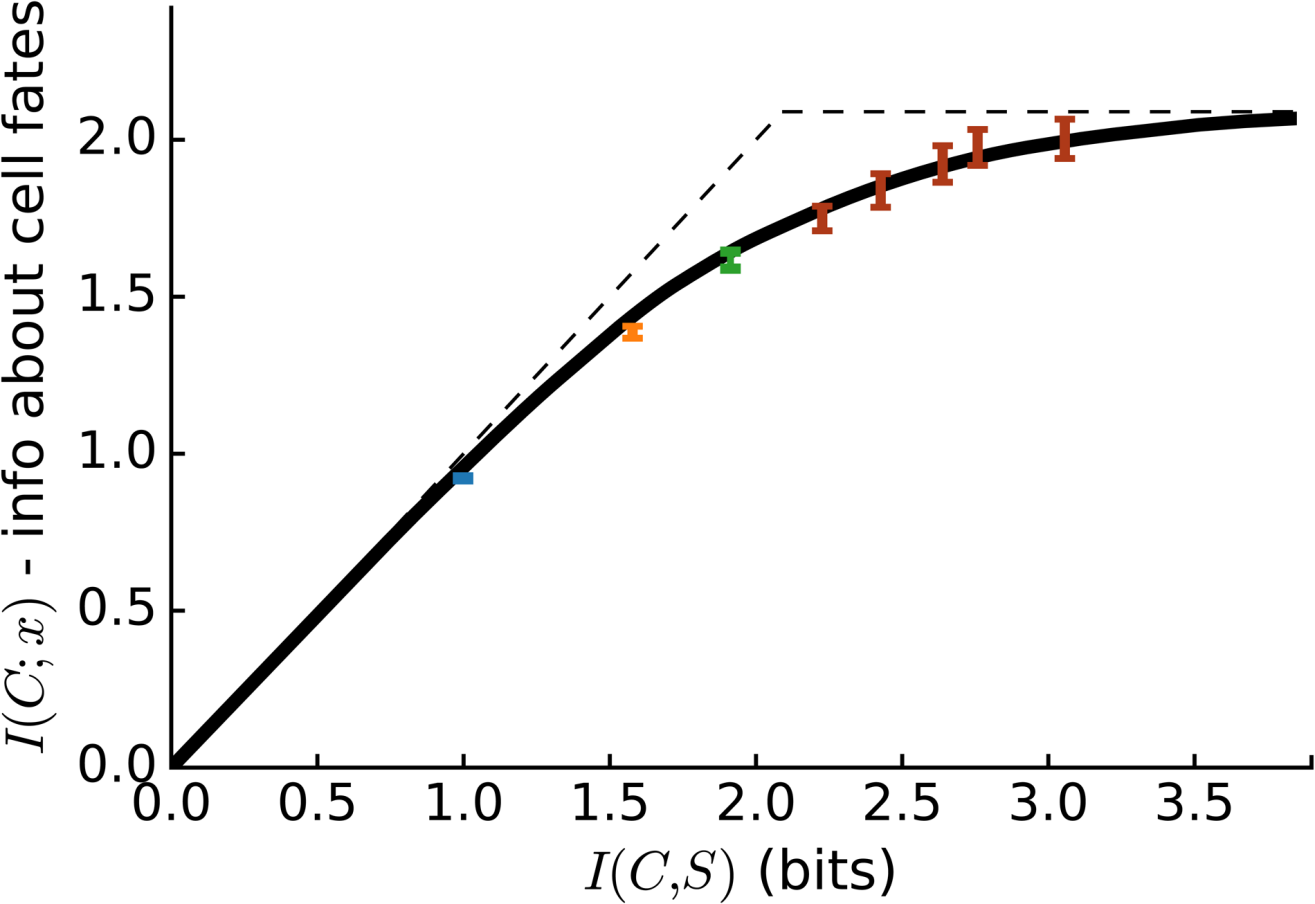
**The bottleneck curve for an optimal sensor for the single protein Hb.** The dashed lines correspond to the data processing inequality, and separate inaccessible from accessible regions of the information plane. An abstract sensor that measures with one (blue), two (orange), three (green) and more (red) thresholds is also on this curve and thus also information-theoretically optimal. Data replotted from [68].

What can one infer mechanistically about how enhancers need to sense these gap transcription factors, if they sensed optimally? To do this, one can either compare the optimal information bottleneck curve to calculations from various mechanisms, or calculate where on the optimal curve various sensors would be. To simplify the question about mechanism, we can use a single gap transcription factor Hb: we imagine that cells need to infer theire fate (or position) from the concentration of Hb, and ask how much information an optimal sensor C can infer given a limit on its capacity I(C;S). Figure 6 shows the optimal bottleneck curve for Hb (with a lower maximum, at I(Hb,X)=2.1 bits, as it is this time only a single transcription factor that is analyzed for cell fate decision making). We compare this optimal curve to the threshold model by optimizing the position of one, two and several thresholds to maximize the information I(θ;X), where θ is a thresholded variable. These thresholds lie exactly on the bottleneck curve. This is important because it means that thresholded measurements of transcription factors, which only trigger when transcription factors concentrations are above or below values, while not mechanistically feasible, are information-theoretically optimal. Thus, the biological intuition that led to the suggestion that the important features of the gap genes were their boundaries is information-theoretic intuition; we can thus make mathematically precise intuition that biologists had for several decades, and expand on it.

Further analysis of these thresholded measurements showed that the threshold positions did not need to be fine tuned: specifically, thresholds at higher transcription factor concentrations could be placed more loosely. Intuitively, this means that in concentration regimes where Hb is expressed noisily, the precise levels are not as important. Biologically, transcription factor concentrations at high concentrations are often measured with weak binding sites. We deduced that this suggests that how many weak binding sites an enhancer has does not matter as much; this, again, is known in biology.

Second, we see that about 10 thresholds are required to sense hunchback correctly. When we use realistic estimates for how well a single enhancer can sense in a Hill-function model with the Berg–Purcell noise (equation 4), we obtained 1–3 bits. While this may just be enough information to sense Hunchback correctly, it is not enough when we want to obtain information about all four gap transcription factors together: there, we needed about 3.8 bits or ca 50 thresholds to get to an accuracy that gets to about 10% of the information provided. This shows that many enhancers are required to read the gap transcription factor signals.

Finally, in order to determine what enhancer architectures should look like if they sensed optimally, one can perform a comparative calculation. We optimized four separate sensors with the constraint that each sensor should only sense one gap transcription factor each. We found that this was always worse than having a single sensor that sensed them together (see blue line in Figure 5). This means that having four enhancers, one of which would sense a single transcription factor, would not be information-theoretically optimal. Indeed, we know that the enhancers that sense the gap transcription factors do have binding sites for many of them at the same time; for example, the Eve stripe 2 enhancer has binding sites for the gap proteins Hb, Kr and Gt [70].

## Perspectives

- We were able to apply a sensing approach to transcription data and found that this captured several aspects of the transcriptional architecture for this network: multiple enhancers which measure gap proteins together (in combinations of expression levels that cannot easily be separated) and with degeneracies for weak binding sites allow the fly to extract most of the protein signal that is provided in the gap transcription factors, and this, in turn, allows the fly to make the correct cell fate decisions.
- The hope is that sensing or inference approaches can, together with mechanistic approaches, help us understand faster why certain regulatory features are there; this could be important not only for a better *in vivo* applications, but also for an appreciation of the regulatory complexity.
- Future directions: Especially for synthetic gene regulation, where one hopes to engineer gene regulatory systems [71–73], often unforeseen bottlenecks arise (see e.g. [74]). A conceptual framework that can identify how important various transcription factor signals are and how they might be sensed in natural systems could help to transfer ideas to the synthetic systems, or help identify what is different.

## Abbreviations

LLPS: liquid–liquid phase separation
LMCs: large monopolar cells
